# Schwannoma in Sellar Region Mimics Invasive Pituitary Macroadenoma: Literature Review With One Case Report

**DOI:** 10.1097/MD.0000000000002931

**Published:** 2016-03-07

**Authors:** Xiangyi Kong, Huanwen Wu, Wenbin Ma, Yongning Li, Yi Yang, Bing Xing, Junji Wei, Yong Yao, Jun Gao, Wei Lian, Zhiqin Xu, Wanchen Dou, Zuyuan Ren, Changbao Su, Renzhi Wang

**Affiliations:** From the Department of Neurosurgery (XK, WM, YL, YY, BX, JW, YY, JG, WL, ZX, WD, ZR, CS, RW); Department of Pathology (HW), Peking Union Medical College Hospital, Chinese Academy of Medical Sciences, Beijing, China.

## Abstract

In central nervous system, schwannomas, as ubiquitous tumors, mostly originate from sensory nerves like auditory and trigeminal nerves. However, intrasellar schwannomas are extremely rare. They are often misdiagnosed as pituitary adenomas.

We report a rare case of schwannoma in the sellar region—a challenging diagnosis guided by clinical presentations, radiological signs, and postoperative pathological test.

We represent a 65-year-old woman who had suffered from headaches, hypothyroidism, and visual disturbance. Her MRI revealed an abnormal sellar region mixed-signal mass lesion with suprasellar, left parasellar, and sellar floor invasiveness. We present detailed analysis of the patient's disease course and review relevant literatures. Written informed consent was obtained from the patient for publication of this article. A copy of the written consent is available for review by the editors of MEDICINE. Because this article does not involve any human or animal trials, there is no need to conduct special ethic review and the ethical approval is not necessary.

When surgically treated, her specimen revealed a typical histopathology pattern of schwannoma. The patient's symptoms improved a lot after surgery and he continues to be under observation.

Despite its rarity, intrasellar schwannoma should be considered in the differential diagnosis of sellar lesions that mimic pituitary adenomas.

## INTRODUCTION

Schwannomas mostly originate from sensory nerves like auditory and trigeminal nerves in the nervous system, and account for 8% to 10% of all primary intracranial tumors. However, they are uncommon in the pituitary sellar region. The most common tumor type in the pituitary fossa and parasellar area is the pituitary adenoma, whereas schwannomas are among the rarest. In fact, only 24 cases of intrasellar schwannoma have been reported in the literature up to now.^1^ Partly because of the diversity and visual similarity of intrasellar or perasellar lesions, neurosurgeons and endocrinologists are often hard-pressed to make accurate preoperative diagnoses. Here, we present an unusual case of schwannoma in the sellar region in a patient who presented with symptoms of headache, hypothyroidism, and visual disturbance. On MRI, the mass was signal-mixed with suprasellar, left parasellar, and sellar floor invasiveness, initially indistinguishable from an invasive pituitary macroadenoma.

## CASE REPORT

A 65-year-old woman came to PUMCH with headaches, fatigue, feebleness and depression, and visual disturbance of 4 months’ duration, which had worsen since the previous 2 weeks. She denied polydipsia, polyuria, sexual hypoactivity, or any symptoms of unconsciousness, epilepsy, convulsion, and cognitive disorders. Physical examinations revealed that her left visual acuity was 0.8 and the right was 0.6. Goldmann perimetry revealed a temporal hemianopia for the left eye and some severe temporal scotomas for the right eye. Other neurological examination results were normal. Her history was negative for head trauma. His social and family history and his system review were negative.

**FIGURE 1:**
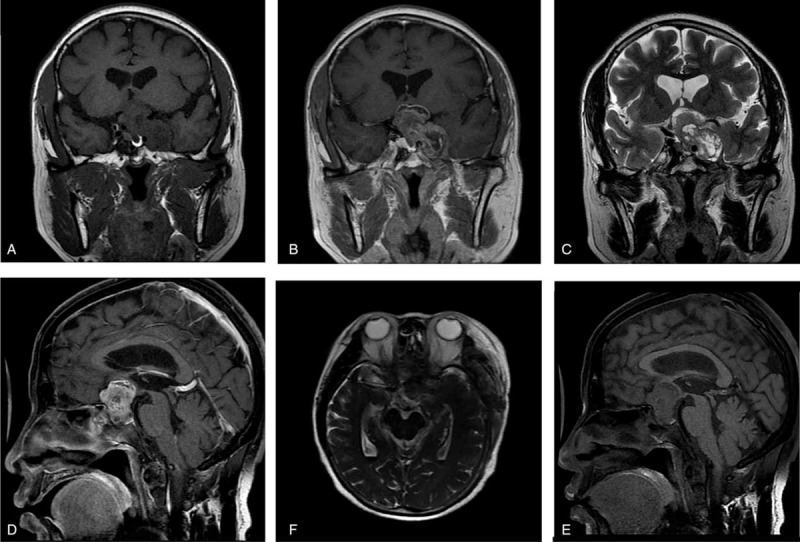
MRI showed an abnormal sellar region: a mixed-signal lesion with suprasellar, left parasellar, and sellar floor invasiveness. (A) coronal T1WI; (B) coronal enhanced-T1WI; (C) coronal T2WI; (D) sagittal T1WI; (E) sagittal enhanced-T1WI; and (F) axial T2WI.

Her MRI revealed an abnormal sellar region mixed-signal mass lesion with suprasellar, left parasellar, and sellar floor invasiveness (Figure 1). The lesion was about 3.6 × 3.4 × 2.7 cm. Equal T1 and T2 signals dominated in the peripheral part of the lesion; with multiple-sheet long T1 and long T2 signals were inside the lesion. A dynamic contrast-enhanced scan showed obvious peripheral inhomogeneous enhancement. Relatively normal pituitary tissue with normal enhancement could be seen near the inferior lesion margin, but was squashed toward the right. The optic chiasma was obviously invaded and compressed. The left cavernous sinus was completely wrapped with right shift and the right cavernous sinus was partly wrapped. Sphenoid sinus mucosa was thickened and showed marked enhancement. Based on these radiological features, our initial diagnosis was an invasive pituitary macroadenoma.

As the patient's fatigue, weakness, and depression were most likely caused by anterior pituitary function damage, or more specifically a hypothyroidism, we performed a series of endocrine tests (Table 1), which indicated a pituitary hormone disorder (including hyperprolactinemia and secondary hypothyroidism) induced by the pituitary lesion.

**TABLE 1:**
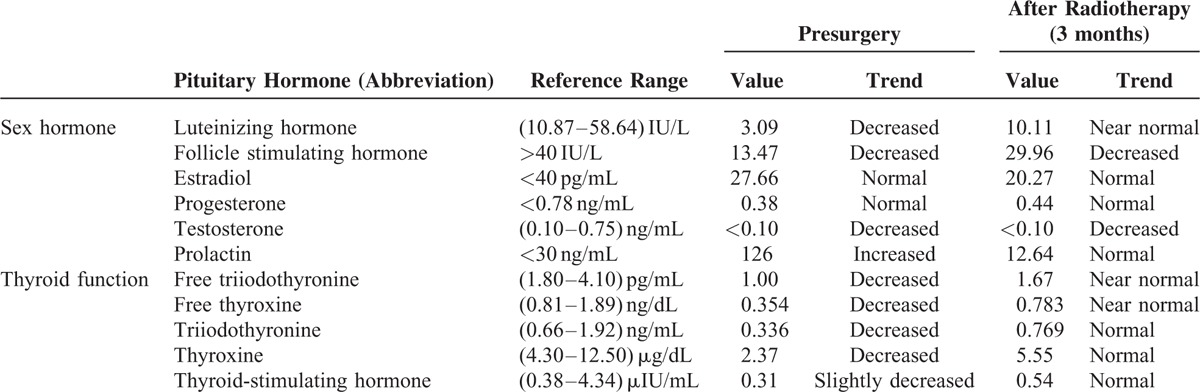
Results of Endocrine Studies for the Pituitary Gland Before and After Surgery

**FIGURE 2:**
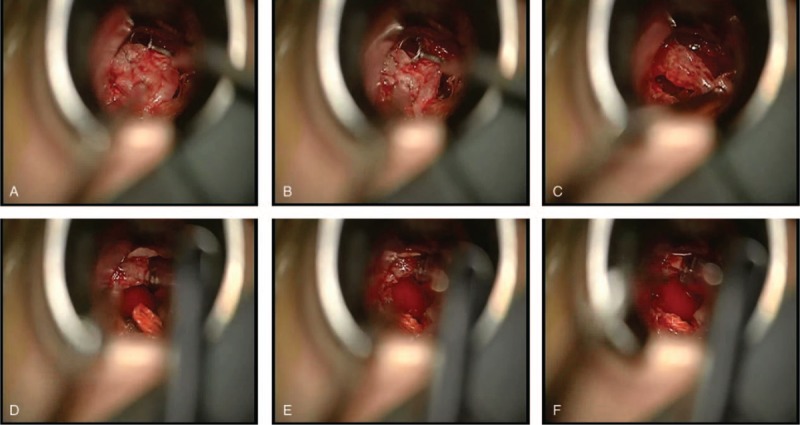
Some intraoperative photographs of the schwannoma during microsurgery. Subtotal resection was achieved eventually.

She then underwent a surgical exploration through a transsphenoidal approach. After drilling the sellar floor and opening the dura, a firm, tough, wheaten mass was found. As its consistency was too rubbery to be easily cut by a surgical blade, and it adhered so tightly to the left cavernous sinus and carotid, only subtotal resection was ultimately achieved. Some intraoperative photographs of the tumor during surgery are seen in Figure 2. Repair of the sellar defect was done with autologous fat and fascia lata.

**FIGURE 3:**
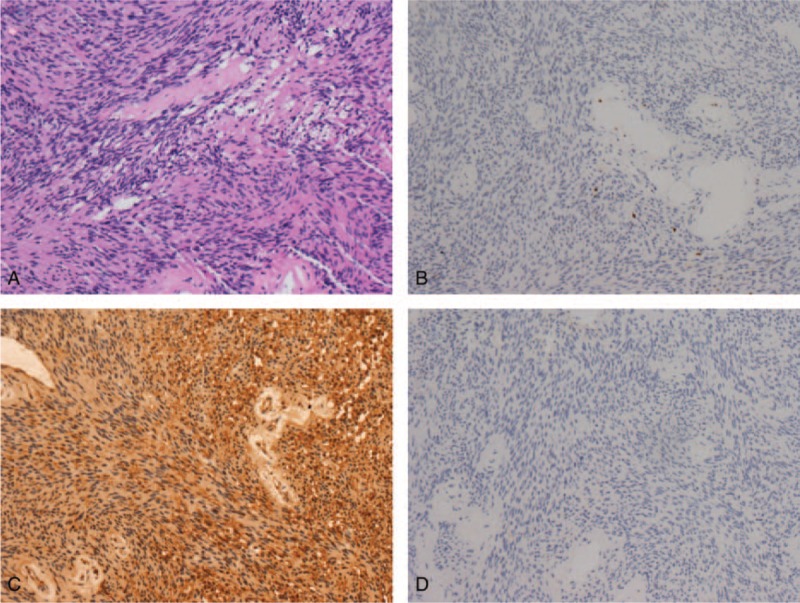
(A) Typical pattern of schwannoma spindle cells shown in pathologic specimen. (H&E ×100). (B) Ki-67 proliferation index of approximately 2% (×200). (C) Positive immunohistochemical staining for vimentin protein (×100). (D) Negative immunohistochemical staining for human epithelial membrane antigen (EMA) (×100).

Against all expectations, a micrograph of the surgical specimen recommended its diagnosis as a schwannoma, revealing multiple portions of stromal-type tissue consisting of spindle-shaped cells with eosinophilic wavy cytoplasm and elongated nuclei. The Ki-67 proliferation index was approximately 2%. Immunohistochemical staining was positive for vimentin protein; immunohistochemistry was negative for human epithelial membrane antigen and S-100 protein. Together, these findings confirmed the schwannoma diagnosis (Figure 3).

**FIGURE 4:**
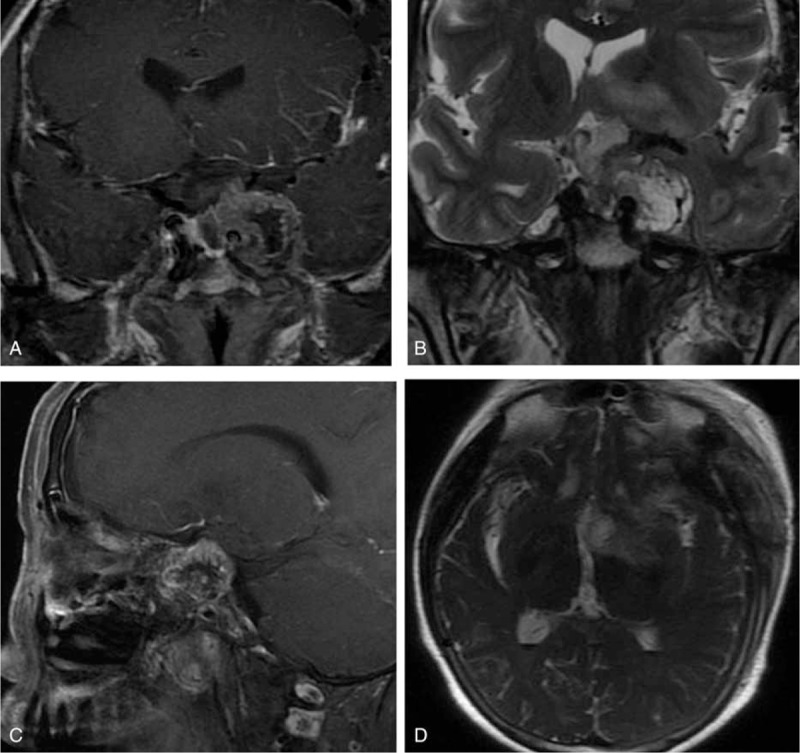
Postoperative MRI of the sellar region showed subtotal resection of the schwannoma. (A) coronal-enhanced T1WI; (B) coronal T2WI; (C) sagittal enhanced-T1WI; (D) axial T2WI.

The patient reported no side effects of surgery and was discharged from hospital 8 days afterwards in good clinical condition. The postoperative period was uneventful. An MRI of the sellar region 3 months after surgery showed subtotal resection of the tumor (Figure 4). Her headache was completely cured, and her visual field relieved somewhat: Goldmann perimetry of her left eye showed a left homonymous inferior quadrantanopia and some left homonymous superior scotomas; the right scotomas was decreased by half. Her activities of daily living and work were not affected. Pituitary functions were also greatly improved despite slight abnormality by 3 months’ follow-up (Table 1). The patient continues to be under observation.

## DISCUSSION

Pituitary adenomas comprise the overwhelming majority of primary tumors in sellar region. The remaining neoplasms include other tumors of pituitary origin (pituitary adenoma, pituicytoma, adenocarcinoma, and pituitary stalk astrocytoma), tumors of nonpituitary origin (chordoma, meningioma, glioma, germ-cell tumors, craniopharyngioma, lipomas, chondromas, leiomyoma, myxoma, and metastatic disease mainly from breast and lung cancers), and other uncommon conditions (cysts, abscesses, arteriovenous fistulas, lymphocytic hypophysitis).

Usually, differential diagnoses of sellar, parasellar, and suprasellar lesions include pituitary adenomas, craniopharyngiomas, meningiomas, and cholesteatomas alike. Schwannomas, however, are not commonly included, because of their exceeding rarity in this region, although they represent approximately 8% of all intracranial neoplasms.^2,3^ The origin of primary schwannomas in the sellar region is still unclear. As the sellar region has no obvious nerve, some authors hypothesized that tumors originate from lateral sellar nerve plexus, perivascular schwann cells adjacent to the medial wall of the pituitary fossa, or sensory nerves of the dura.^2^ Studies to elucidate the histopathogenesis of these neoplasms are warranted.

Intracranial schwannomas can occur sporadically or present as manifestations of central neurofibromatosis (type 2), which are most often found in young males.^2^ Few cases of real primary intrasellar schwannomas have been reported previously. Recently, Sharifi et al performed a literature review of intrasellar schwannomas reported before, all of which presented a suprasellar or parasellar extension similar to that of the present case.^1^

Generally, the anatomical location determines the clinical features of intrasellar schwannomas. Those located within the sella turcica typically present with symptoms similar to pituitary adenomas.^4^ Lateral expansion into cavernous sinus may compress oculomotor, trochlear, and/or abducent nerves, leading to diplopia and then ocular misalignment. Suprasellar expansion often presents with visual disturbances because of compression of the optic nerve or chiasm; similar to our patient, who developed headaches, and bitemporal hemianopia. When the schwannoma compresses the normal pituitary gland, pituitary hormone disorders may follow. Interestingly, most of these cases showed hypopituitarism.^4^Table 2 summarizes 9 previously reported intrasellar schwannomas with hypopituitarism.^5–13^ In our case, the pituitary dysfunction was hypothyroidism and hyperprolactinemia, probably occurring as a result of the compression against the hypothalamic-pituitary stalk. To our knowledge, this is the first intrasellar schwannoma case with such a hormone disorder.

**TABLE 2:**
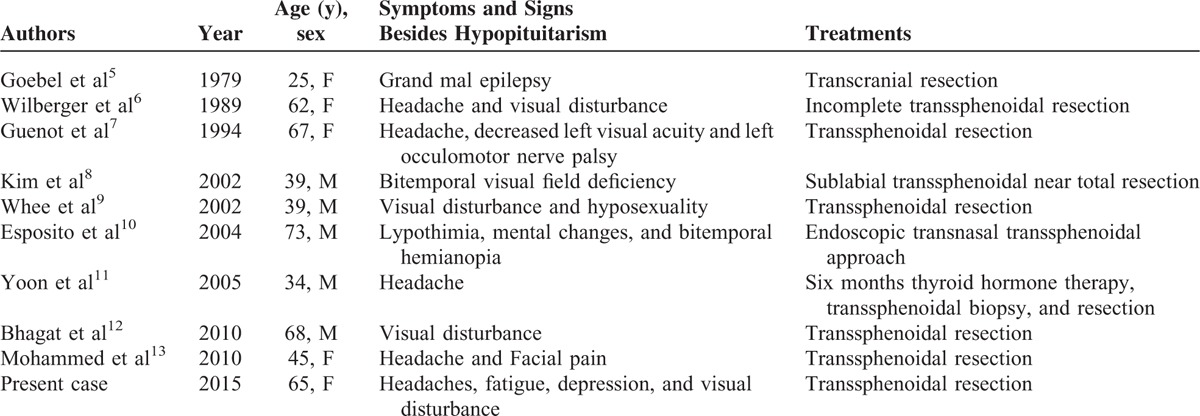
Clinical Review of 9 Previously Published Intrasellar Schwannoma With Hypopituitarism Before Surgery

As with pituitary adenomas, intrasellar schwannomas often present as a mass lesion with an iso-to-hypointense signal on T1WI with homogeneous contrast enhancement; whereas on T2WI, they commonly show a more hyperintense signal than pituitary adenomas. In a study conducted by Sharifi et al,^1^ the MRI findings of a intrasellar schwannoma showed the lesion was hard calcified. In our case, the MRI signals were quite mixed, indicating the internal components were complex and various.

The definitive treatment for intrasellar schwannoma is surgical excision, particularly for those with atypical radiological and clinical manifestations and hard-to-confirm diagnoses.^4,14^ The radiological features dictate the surgical approach by revealing the location and extent of the tumor spread. A microscopic transsphenoidal approach is highly recommended, in that it renders good visualization, facilitates complete or near-complete removal of the tumor possible, and can be technically simpler if the mass has already widened and invaded sinus walls without high morbidity.^4^ Correct diagnosis is usually established by histopathological and immunohistochemical examination of surgical specimens^15^; most reported intrasellar schwannomas were initially misdiagnosed as pituitary adenomas or other tumor types before surgery.^1,9,16^ Although none of the previous reported cases underwent second surgeries for remnant or recurrent tumors, adjuvant treatment such as radiotherapy can be considered.

## CONCLUSION

Although intrasellar schwannomas are not common, they should be considered in diagnosing sellar lesions that mimic pituitary adenomas or meningiomas, especially when the final diagnosis of these rare sellar masses relies on pathology and immunohistochemistry results.
